# Modeling of protein complexes in CASP14 with emphasis on the interaction interface prediction

**DOI:** 10.1002/prot.26167

**Published:** 2021-07-05

**Authors:** Justas Dapkūnas, Kliment Olechnovič, Česlovas Venclovas

**Affiliations:** ^1^ Institute of Biotechnology Life Sciences Center, Vilnius University Vilnius Lithuania

**Keywords:** binding sites, docking, homology modeling, interface scoring, model quality assessment, PPI3D, protein complexes, protein‐protein interactions, template‐based modeling, VoroMQA

## Abstract

The goal of CASP experiments is to monitor the progress in the protein structure prediction field. During the 14th CASP edition we aimed to test our capabilities of predicting structures of protein complexes. Our protocol for modeling protein assemblies included both template‐based modeling and free docking. Structural templates were identified using sensitive sequence‐based searches. If sequence‐based searches failed, we performed structure‐based template searches using selected CASP server models. In the absence of reliable templates we applied free docking starting from monomers generated by CASP servers. We evaluated and ranked models of protein complexes using an improved version of our protein structure quality assessment method, VoroMQA, taking into account both interaction interface and global structure scores. If reliable templates could be identified, generally accurate models of protein assemblies were generated with the exception of an antibody‐antigen interaction. The success of free docking mainly depended on the accuracy of initial subunit models and on the scoring of docking solutions. To put our overall results in perspective, we analyzed our performance in the context of other CASP groups. Although the subunits in our assembly models often were not of the top quality, these models had, overall, the best‐predicted intersubunit interfaces according to several accuracy measures. We attribute our relative success primarily to the emphasis on the interaction interface when modeling and scoring.

## 1 INTRODUCTION

In recent years the progress in three‐dimensional (3D) protein structure prediction was impressive.^1^ Application of deep learning‐based methods now allows modeling of structures for most of the individual proteins.^2^, ^3^, ^4^ However, the majority of proteins do not function in isolation. They usually perform their functions by interacting with other proteins and assembling into stable or transient protein complexes. Therefore, if we wish to have a detailed understanding of how proteins function, the knowledge of the structures of individual proteins is not sufficient. We need to know the structures of corresponding protein complexes.

The number of possible binary protein‐protein interactions is much higher than the number of proteins encoded in genomes, and only a small part of these interactions has already been discovered experimentally.^5^, ^6^ Similarly, the number of different structural types of protein complexes is predicted to be much higher than the number of protein folds.^7^, ^8^ Therefore, the structural modeling of protein‐protein interactions represents a more complex problem than the prediction of structures for individual proteins.

Currently, template‐based modeling and docking are the two main methods used for modeling protein complexes. Template‐based modeling is based on the observation that homologous proteins often interact in the same way.^9^ Thus a known structure of a protein complex can serve as a template for modeling homologous protein complexes. If there are no templates, protein‐protein docking methods can be used.^5^, ^10^ Docking methods aim to find how proteins interact with each other starting from known structures of individual subunits that can be either solved experimentally or obtained by computational modeling.

The field of protein structure prediction is monitored in the Critical Assessment of Structure Prediction (CASP) experiments that explore every aspect of protein structure modeling.^1^ The Critical Assessment of PRedicted Interactions (CAPRI) experiments are devoted to the prediction of the structures for diverse protein complexes.^11^ Both CASP and CAPRI are based on blind testing. The participants are given the sequences of proteins, for which structures are solved experimentally but not published (termed “targets”), and then they are asked to provide structural models. Subsequent comparison of models with the experimental structures enables establishing the current state‐of‐the‐art in the field and also objective comparison of different methods. In recent years, CASP and CAPRI experiments are collaborating in the area of structural modeling of protein complexes,^12^, ^13^ and a category dedicated to assessment of multimeric proteins has been established in CASP as well.^14^, ^15^

We participated in recent CASP and CAPRI experiments, aiming to test our abilities to predict structures of protein complexes using template‐based modeling and free docking.^16^, ^17^, ^18^ Our results demonstrated that there is room for improvement in both of the methods. In template‐based modeling, the identification of templates can be enhanced. In docking, the assessment and selection of correct interfaces from thousands of diverse docking models is probably the most important problem. It is also interesting to see how the progress in protein structure prediction influences modeling of protein complexes. At present, it is often possible to generate sufficiently accurate models of individual proteins, but does this help to predict the protein‐protein interfaces?

To explore these questions in detail, we participated in the CASP14 experiment, where our group (“Venclovas”) performed relatively well, particularly in the interface prediction. In this article we describe our modeling methods and analyze our results in detail aiming to understand what went right, what went wrong and why.

## 2 METHODS

### 2.1 Modeling outline

**FIGURE 1:**
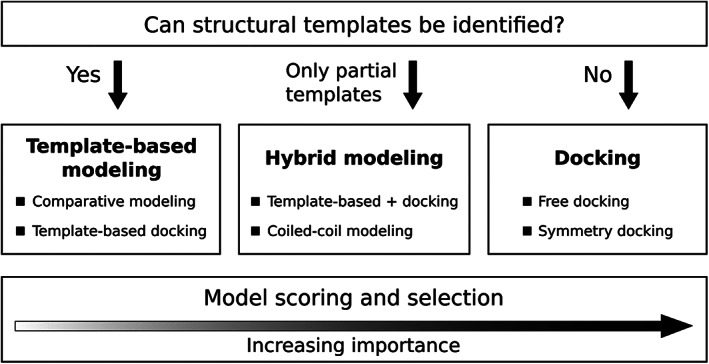
Summary of the “Venclovas” group modeling workflow in CASP14

The outline of our modeling workflow in CASP14 is shown in Figure 1. During the initial step, for every target we attempted to identify multimeric templates for comparative (homology) modeling using sequence‐ and structure‐based search methods and to generate models of the whole protein complex. If we could identify templates, but the sequence‐structure alignments were not reliable, we then used template‐based docking, that is, we aligned monomer models to the chains of the multimeric template to obtain a multichain structure. Coiled‐coil protein structures were predicted using a custom‐designed procedure. If no templates could be identified, we switched to free docking. In the case of large multisubunit targets, we combined all the methods, employing a comparative modeling approach for the parts of the complex for which templates were available followed by docking of the resulting subcomplexes to obtain the full assembly. Compared to CASP13,^18^ the major modifications of our modeling pipeline included the introduction of structure‐based searches for multimeric templates, the use of a novel model selection protocol emphasizing the interface scores, and the application of short molecular dynamics simulations to rigid‐body docking models in order to improve the geometry of interchain interactions.

### 2.2 Comparative modeling

The outline of multimeric comparative modeling pipeline is provided in Supplementary Figure S1. Starting with target sequences, we first searched for potential templates using PPI3D^19^ and HHpred^20^, ^21^ web servers. If this step failed to identify reliable templates (HHpred probability ≥90%) and/or produced incomplete alignments, we additionally employed structure‐based searches. In this case, selected monomeric CASP server models corresponding to the subunits of a target protein complex were used as queries for PDB searches using the DALI server.^22^ The aim was to identify multimeric PDB assemblies that could potentially serve as multimeric templates. Once templates were identified using either sequence‐ or structure‐based searches, structural models for the whole protein complex were generated by MODELLER^23^ and its plugin AltMod^24^ using the multichain modeling function. For the simpler cases, where the use of PPI3D server sufficed, comparative modeling was automatic, except for the choice of structural templates and for the assessment and ranking of models based on different templates.

### 2.3 Template‐based docking

For some targets neither sequence‐based nor structure‐based searches produced alignments with multimeric homologs of sufficient reliability and/or coverage such that these alignments could be used for multimeric comparative modeling. However, in some cases we observed that the proteins identified as potential multimeric templates had similar functional annotations as the CASP targets. If the alignment of CASP server models to these multimeric structures using TM‐align^25^ seemed plausible, we constructed assembly models by simply aligning monomeric CASP server models to different chains of the templates and then relaxing the resulting models to remove steric clashes using the same methods as in the case of free docking.

### 2.4 Coiled‐coil modeling

Several targets were predicted to be coiled coils using MultiCoil2^26^ and similar sequence analysis tools provided in MPI Bioinformatics Toolkit.^20^ Such targets were modeled using a custom‐designed procedure: structure models were automatically generated by MODELLER/AltMod using the same manually selected coiled‐coil template and automatically generated all possible gapless target‐template alignments followed by model selection.

### 2.5 Free docking

When no templates could be found for protein complexes, free docking of top five selected monomeric CASP server models was done by Hex^27^ for hetero‐complexes and Sam^28^ for homomultimers (Supplementary Figure S2). The resulting models were ranked using both global and interface VoroMQA scores as described below. Next, top 100‐500 models were relaxed by a very short molecular dynamics simulation using OpenMM software,^29^ Amber99SB force field, and GBSA‐OBC solvation model.^30^, ^31^ The relaxed models were subsequently re‐ranked using the same scoring procedure and clustered according to the interface Contact Area Difference score (CAD‐score)^32^, ^33^ values aiming to select a diverse set of models. The free docking workflow was fully automated, but the final models were always inspected visually.

### 2.6 Hybrid modeling

For some large target protein complexes structural templates were available only for some of the subunits or domains. In these cases, a hybrid modeling strategy was used, that is, part of the complex was modeled using comparative modeling, whereas remaining subunits were docked to it either by template‐based docking using TM‐align^25^ or by free docking. In addition to that, a mixture of free docking and template‐based models was submitted for several smaller targets that had templates only from structure‐based search.

### 2.7 Model selection

For model selection we used both global structure scores and interface scores. This approach was described previously^16^, ^18^ and implemented in the VoroMQA web server,^34^ but for CASP14 we introduced some modifications. These included VoroMQA‐dark, a new method for global structure evaluation (see below), and an improved tournament‐based ranking algorithm (see Supplementary Information for details). When ranking models based on their pairwise comparisons, the algorithm puts more emphasis on the interface pseudo‐energy and less emphasis on the global structure score. This is achieved by using a tolerance value when comparing global VoroMQA‐dark scores. If the difference between global scores is small, the interface scoring becomes the only decisive factor. We named the new VoroMQA‐dark‐based model selection protocol as “VoroMQA‐select‐new.” In addition to fully automated scoring methods, models were also evaluated according to constraints obtained from the literature or from the CASP contact prediction servers, if such data were available. All models were visually inspected before submission and manual ranking adjustments were introduced, if necessary. These manual adjustments were predominantly applied in the hybrid modeling cases.

### 2.8 VoroMQA‐dark method for model quality assessment

VoroMQA‐dark is a new model quality assessment method based on the previously published VoroMQA^35^ method (which will be referred to as VoroMQA‐light). VoroMQA‐dark uses a neural network (NN) trained to predict local (per‐residue) CAD‐score^32^ values. The global structure score is computed by averaging the predicted local scores. The NN input vector for each residue is computed from the Voronoi tessellation‐based contact areas and the corresponding contact potential values from VoroMQA‐light. See Supplementary Information for more details on VoroMQA‐dark. The VoroMQA‐dark standalone software is included in the extension of the Voronota^36^ package freely available from https://kliment-olechnovic.github.io/voronota/expansion_js/.

## 3 RESULTS

### 3.1 Overview of the results

To analyze our performance in CASP14 we used several accuracy measures designed to evaluate various features of multimeric models. For the overall model evaluation we used QS‐score, a distance‐based measure of interface accuracy.^37^ To make qualitative model accuracy assignments we converted QS‐score values to CAPRI‐like accuracy categories.^38^ Four other scores were used to assess the interface and the overall structure accuracy. Interface Contact Similarity (ICS or F1‐score) and Interface Patch Similarity (IPS or Jaccard coefficient) were used to assess contact and interface patch prediction, respectively.^14^ Oligomeric lDDT and TM‐score were used to assess overall structure accuracy. lDDT is an all‐atom superposition‐free score,^39^ whereas TM‐score is based on the rigid body superposition of Cα atoms.^40^, ^41^ In addition to the above scores reported by the Prediction Center, we also used CAD‐score^32^, ^33^ to evaluate both structure and interface accuracy.

The summary of our modeling results based on the QS‐score is presented in Table 1, whereas the detailed accuracy evaluation of our best models is provided in Supplementary Table S1. As can be seen in Table 1, for 11 targets we identified multimeric templates and used comparative modeling, producing medium or high‐accuracy models for eight of them. In the absence of reliable target‐template sequence‐structure alignments, we applied template‐based docking using TM‐align. This approach resulted in models of medium accuracy for two targets. Hybrid approaches utilizing both comparative modeling and docking steps were used for nine targets with relative success. The results of free docking were ranging from completely incorrect to medium‐accuracy models. The custom modeling procedure that we used for coiled coils did not produce any reliable models.

**TABLE 1:** Summary of the “Venclovas” group CASP14 assembly modeling results

Modeling strategy	Number of targets	Accuracy category of our best model			
		High (QS‐score ≥0.7)	Medium (0.3 ≤ QS‐score < 0.7)	Low (0.1 ≤ QS‐score < 0.3)	Incorrect (QS‐score <0.1)
Comparative modeling	11	1	7	0	3
Template‐based docking	2	0	2	0	0
Hybrid	9	1	3	5	0
Free docking	5	0	1	2	2
Coiled‐coil modeling	2	0	0	1	1
Total	29	2	13	8	6

### 3.2 Modeling results in the context of other CASP14 groups and automated model selection

The results, presented in Table 1 and Supplementary Table S1, do not tell much about our relative success. To investigate our performance in the CASP14 context, we compared our results (group “Venclovas”) with those of three other top‐performing groups for models designated as first (model 1). We also included our automatic model selection protocol (“VoroMQA‐select‐new”) as a virtual group, allowing it to make selections from all CASP14 multimeric models (produced by both automatic servers and human groups). By doing this we aimed to test the effectiveness of our automatic scoring in a scenario where a set of diverse models, generated by multiple methods, is available to select from. For the performance comparison, we used the sum of *z* scores of two interface accuracy measures (ICS and IPS) and two global structure accuracy measures (lDDT and TM‐score).

**FIGURE 2:**
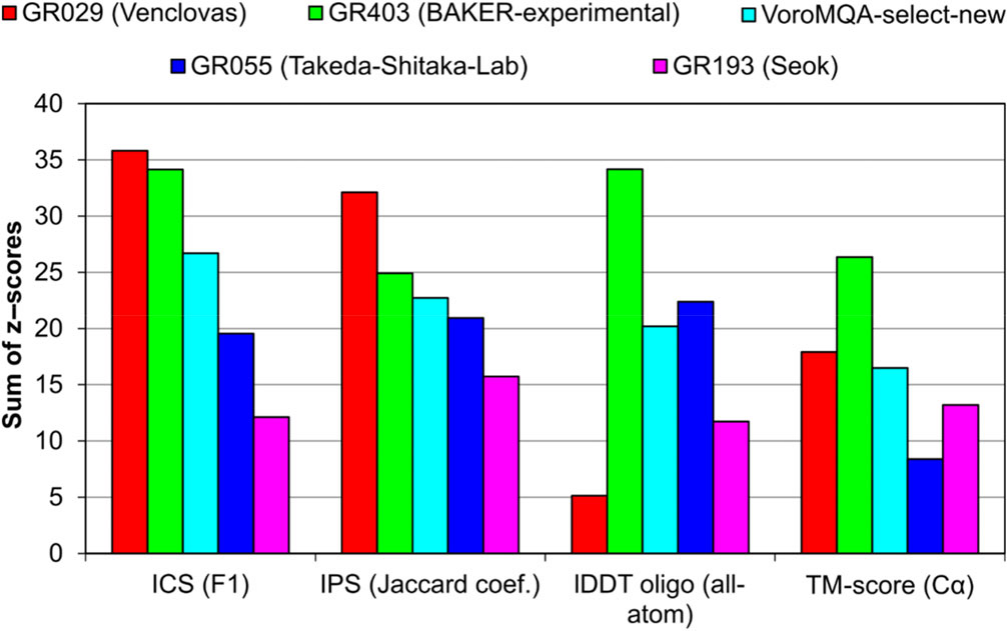
Comparison of results of our group (“Venclovas”) and our automated model selection protocol (“VoroMQA‐select‐new”) with other top‐performing CASP14 groups

The comparison, shown in Figure 2, revealed that different features of our models were predicted with different level of success. According to the accuracy of intersubunit interfaces (ICS and IPS) we achieved the best results. We were particularly successful in predicting interface patches (IPS). On the other hand, the global structure accuracy of our models is not so great compared to other top‐performing groups. This is especially visible if we consider lDDT, an all‐atom score, largely reflecting the accuracy of individual subunits. Interestingly, our automatic model selection protocol showed relatively strong performance, taking the third position by any of the four scores. Although this method performed worse than our human group on both interface accuracy measures and TM‐score, the results according to all‐atom accuracy (lDDT) were quite a bit better.

**FIGURE 3:**
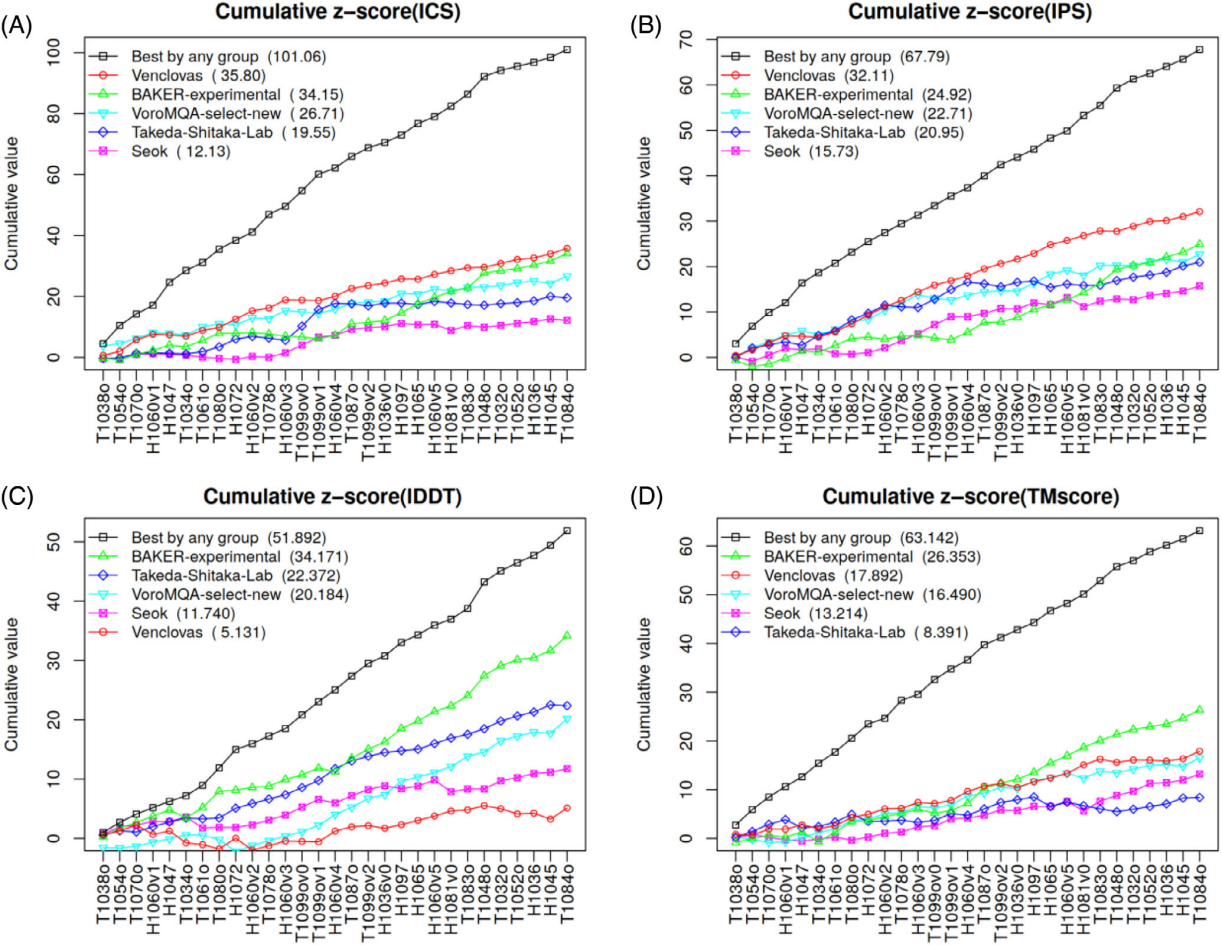
Cumulative *z* score values of the models designated as first. Targets were ordered by the maximum achieved ICS score. Group names in the plot legends are ordered by the corresponding sums of *z* scores that are shown in brackets. ICS, Interface Contact Similarity

To look at different features in more detail, we examined per‐target *z* scores. *z* Score values were accumulated progressively for targets ordered by the maximum ICS value of all the models produced by any group for a given target. Such an ordering may be interpreted as an estimate of the target difficulty. Figure 3 shows the resulting plots for the models designated as first (model 1). In addition to the data for the same top groups and “VoroMQA‐select‐new,” the plots also include the data for the best models provided by any predictor group. The latter curve may be considered as a reference by representing the upper limit of what could have been achieved in CASP14.

Interestingly, the per‐target analysis (Figure 3) revealed that the relative success of different groups was dependent not only on the evaluation measure as seen in Figure 2, but also on the set of prediction targets. According to the interface prediction accuracy, our group dominated for most of the targets [Figure 3(A,B)]. On the other hand, if we consider the global accuracy of models the picture is different. According to TM‐score [Figure 3(D)] our models are below the state‐of‐the‐art for about half of targets, whereas according to lDDT [Figure 3(C)] this is true for nearly all the targets. To see whether our models as assessed by lDDT were indeed significantly inferior to those of other top groups, we examined the cumulative raw values (Supplementary Figure S3). Surprisingly, it turned out that the absolute differences between the groups, especially if evaluated using lDDT (Figure S3F), are relatively small. This indicates that in most cases subunit structures were of comparable accuracy and that relatively large *z* score differences resulted from small structural improvements (see examples in Figure S4). The same analysis performed with the CAD‐score‐based analogs of ICS, IPS, and lDDT scores led to similar conclusions (Supplementary Figure S5).

In addition to individual scores, we analyzed their combinations reflecting either the interface prediction accuracy or the accuracy of both the interface and the global structure. We performed this analysis both for models designated as first (Figure S6) and for the best‐of‐five models (Figure S7). The analysis of these combinations has further corroborated above observations on our relative success in the interface prediction and on target‐dependent group performance. Interestingly, in the analysis of best‐of‐five models our automatic selection protocol (VoroMQA‐select‐new) was the best according to the interface accuracy [Figure S7(A,C)] and close to the top according to the combined accuracy [Figure S7(B,D)]. Although having access to all the models VoroMQA‐select‐new had an important advantage over other groups, the results suggest that this automatic selection procedure is quite robust.

### 3.3 Template‐based modeling

As the structures of protein complexes are often evolutionary conserved,^9^ template‐based modeling is currently the most reliable method to model them. Straightforward multimeric comparative modeling resulted in medium to high accuracy models for 8 of 11 CASP14 targets. Template‐based docking also resulted in medium‐accuracy models for two targets. Thus, if reliable templates were available the template‐based approach worked well for both homomers and heteromers.

Identifying the correct template having the same oligomeric state is the key to successful modeling of protein complexes.^16^, ^17^, ^18^, ^37^ Ambiguous oligomeric state of templates may be the reason why we failed to model T1034, for which we used templates having different oligomeric states.

In CASP14 we had additional examples demonstrating the limitations of template‐based modeling for protein complexes. One such example, H1036, represents a trimeric viral protein bound to an antibody. Our models were based on homologous trimer structures bound to antibodies. This resulted in good models of homotrimer interfaces, but the antibody was bound to a completely different epitope (Figure S8). This incorrectly predicted interface is not surprising bearing in mind the nature of antibody‐antigen interactions. The binding site in the antibody (paratope) is formed by hypervariable loop regions, and the antigen‐binding site (epitope) can be anywhere on the protein surface.^42^

Our results for T1099 show another limitation of template‐based approach for protein complexes. This target is a large viral capsid, yet modeling its structure can be reduced to a problem of predicting a homotetramer (T1099v0) having two different interfaces (T1099v1 and T1099v2). Our models contained high‐accuracy interface 2, yet the interface 1 was incorrect (Figure S9). The reason for this failure was the large insertion in the target interface, compared to the template structures.

### 3.4 Hybrid modeling of large targets

When modeling a large protein complex templates might be available just for some parts of it. During CASP14 this was the case for heteromeric targets H1060 and H1097. For both of these targets more accurate models were generated for those parts that had templates.

**FIGURE 4:**
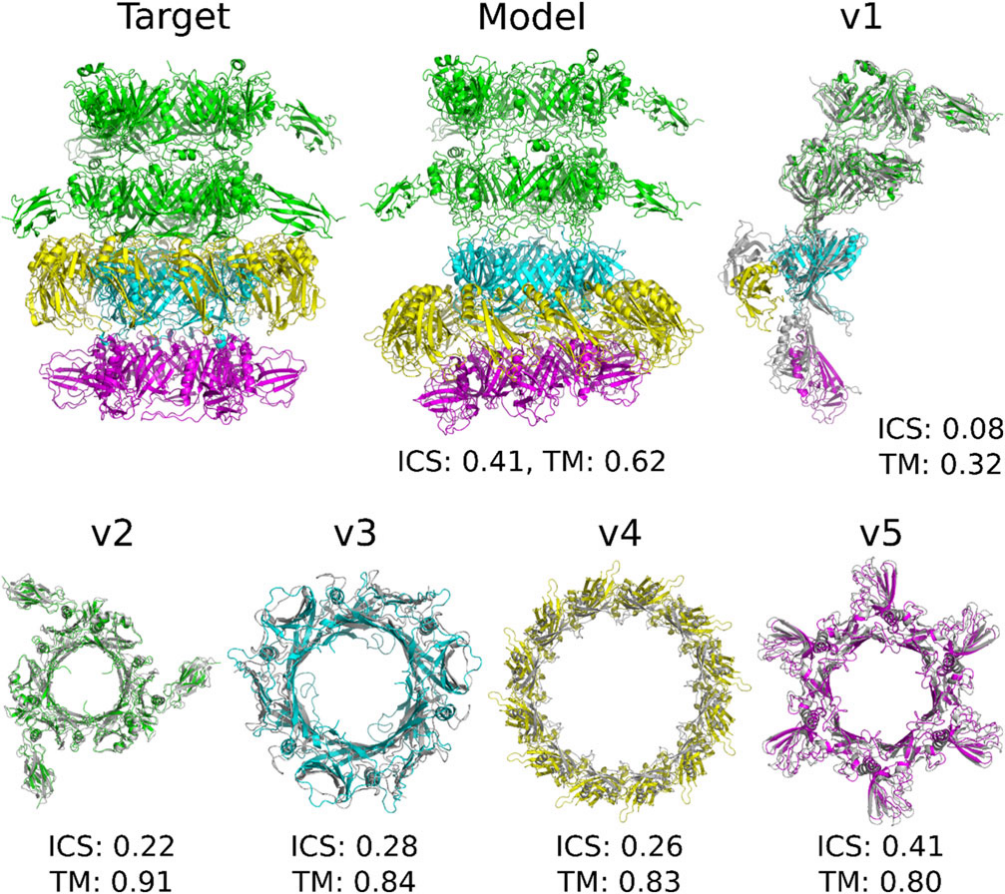
Modeling of H1060. In the images of subcomplexes v1–v5 the target structure is shown in gray and the model structure is colored

H1060 was a viral protein complex containing 27 subunits (five homomeric rings bound to each other, A6B3C12D6, Figure 4). We found templates for all the rings and generated models of medium accuracy for all of them using either comparative modeling or template‐based docking methods. Next, we tried to do template‐based docking of the ring models to each other using another viral template, but for such a large complex the docking was complicated. As a result, the accuracy of hetero‐complex (H1060v1) model is lower than the accuracy of models for individual rings.

Similar situation was observed with hetero‐pentamer H1097, where we tried to dock the domains of the fifth subunit to a homology model of a hetero‐tetramer albeit with limited success (Supplementary Table S2).

### 3.5 Free docking

**FIGURE 5:**
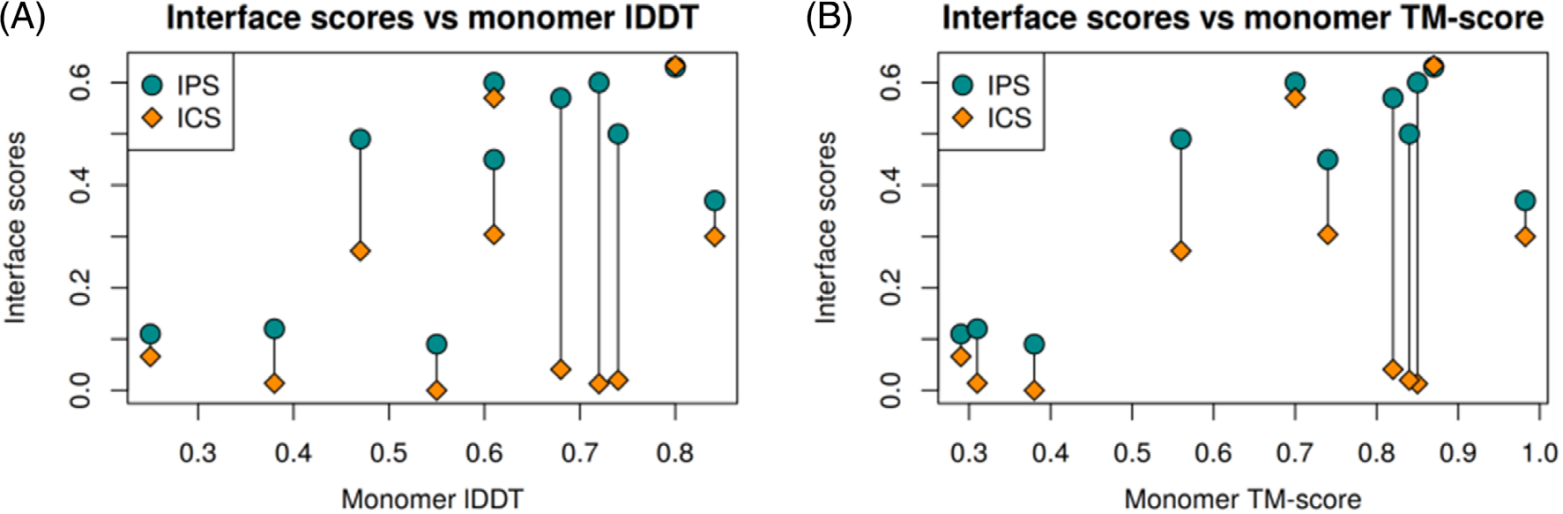
Dependence of the predicted interface accuracy on the subunit accuracy in our free docking models having the largest ICS or IPS scores. ICS, Interface Contact Similarity; IPS, Interface Patch Similarity

Our free docking results show similar trends as in previous CASP and CAPRI experiments (Figure 5; Supplementary Table S3).^18^ First, the IPS values sometimes (in 4 of 11 analyzed cases) are much higher than ICS. This indicates that the residues mediating protein‐protein binding are predicted better than the mutual subunit orientation defining the exact contacts at the interface. Another observation is that the accuracy of subunits matters a lot when docking modeled protein structures. We did not produce any acceptable accuracy models when we were starting from subunit structures of lower accuracy (lDDT <0.4 or TM‐score <0.5). It is also important to note that the opposite is not necessarily true. Accurate models of individual subunits do not guarantee accurate docking models.

**FIGURE 6:**
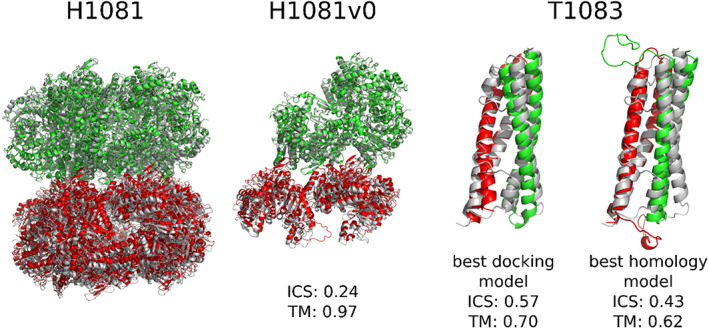
Successful docking models for H1081 and T1083

The most successful docking results were obtained for H1081v0, T1083 (Figure 6), and T1084. Interestingly, in the cases of T1083 and T1084 the free docking models were better than the template‐based models, but the reasons for this are not clear. H1081 was a large target, where two decameric rings had to be docked, and for that we developed a custom procedure. The homology models of decameric rings were aligned on the axis perpendicular to the ring plane and then pushed to each other (using 1 Å steps) and rotated around the axis (using 2° steps), saving every distinct arrangement. Afterward all obtained models were relaxed, scored, and ranked. This custom “two‐ring docking” procedure resulted in surprisingly good models.

**FIGURE 7:**
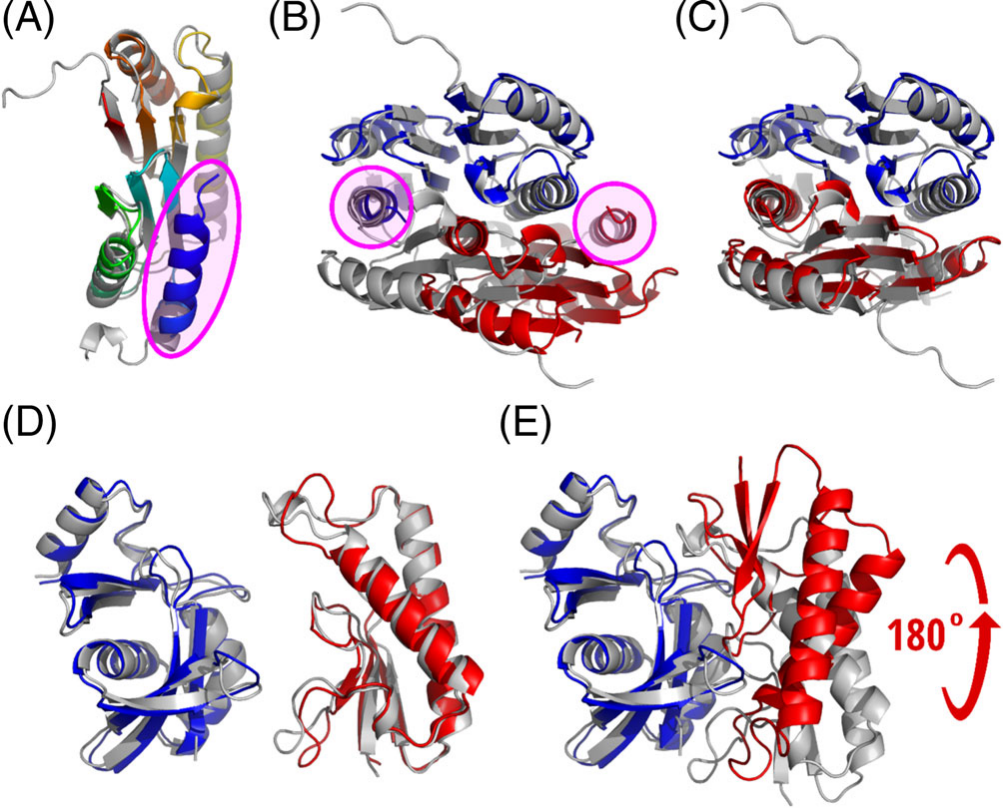
Free docking results for T1054 and H1065; (A) a monomer model of T1054 has additional folded helix in the N‐terminus (blue, encircled); (B) a dimer model (interface CAD‐score 0, binding site CAD‐score 0.34), in which the N‐terminal helix (encircled) occupies the place of another subunit; (C) a dimer model (interface CAD‐score 0.33, binding site CAD‐score 0.58), in which a monomer without N‐terminus was used for docking; (D) monomer models of H1065 (PDB: 7M5F); (E) interface patch is predicted better than the interface contacts for H1065 because subunit 2 (red) is rotated ~180° in the best model of H1065 (ICS = 0.04, IPS = 0.57). Target structures are gray, model structures are colored in all images

The modeling of other docking targets was less successful (Figure 7) illustrating common problems related to the monomer model accuracy and scoring. For example, in the monomer structure of T1054 which we used for docking, the position of N‐terminal helix is not compatible with the dimeric structure. The helix is too well packed against the subunit structure, occupying the place of a helix from another subunit in the dimer [Figure 7(A,B)]. Therefore, it was impossible to obtain a correct docking model starting from such a monomer. Interestingly, when during the post‐CASP analysis we repeated the docking experiment using the same monomer model, but without the N‐terminal helix, the docking was highly successful [Figure 7(C)]. Of note, in solution this protein exists as a mixture of a monomer and a decamer, and the dimer observed in the crystal might represent an intermediate state in decamerization.^43^

In our best model for heterodimeric target H1065 one of the subunits is rotated ~180° compared to the experimental structure [Figure 7(E)]. Again, the interface patch is identified correctly while the interface contacts and subunit orientation are different. Both monomer models are fairly accurate (lDDT >0.65, CAD‐score >0.7, TM‐score >0.8), therefore, their accuracy probably is not the reason for incorrect docking [Figure 7(D)]. However, scoring is really problematic for H1065: both the global and the interface VoroMQA scores of the experimental structure and the model are highly similar (global scores: 0.70 vs 0.68, interface energy: −354 vs −388 for target and model, respectively). In other words, even if the experimental structure was present among the models it would not necessarily have been selected.

## 4 DISCUSSION

During CASP14 our group used well‐known methodologies for structural modeling of protein complexes: template‐based modeling and rigid‐body docking. We did not use any deep learning‐based interchain contacts prediction or refinement using extensive molecular dynamics simulations. Our main aim was accurate prediction of protein‐protein interfaces, even if this meant lower global accuracy of models. As the interface accuracy of our models designated as first was the best among CASP14 groups predicting protein assemblies [Figures 2 and 3(A,B)], it appears that we have coped with this task quite successfully. Probably the main reasons for the successful modeling were (1) effective multimeric template identification by sequence and structure‐based methods, (2) model selection procedure, involving improved VoroMQA scoring with more emphasis on the interaction interface, and (3) short molecular dynamics simulations aimed at removing unrealistic geometry and clashes in docking models.

Unlike the interface accuracy, the global accuracy of our models was not the highest [Figures 2 and 3(C,D)]. This is particularly evident from the close to average values of lDDT, the score that considers all atoms. Most of these lower scores came from template‐based models generated using MODELLER/AltMod. When we used CASP server models for docking, lDDT scores were typically higher. This suggests that the global accuracy of our template‐based models might have been higher had we used more advanced modeling techniques.^44^

The template‐based modeling remains the most accurate method to predict the structures of protein complexes, but the limiting factor for this approach is the detection of structural templates. Typically templates are identified by sequence‐based search methods such as BLAST, PSI‐BLAST,^45^ or HHpred.^21^ In CASP14, aiming to expand the set of available templates, we additionally employed structure‐based searches. The efficiency of structure‐based approach has been greatly increased by the recent advances in protein structure prediction.^3^, ^4^ The availability of more accurate models for monomers may be the reason why our structure‐based template searches successfully complemented sequence‐based searches in CASP14, but less so in CASP13.^18^ It is possible that the structure‐based template identification for protein complexes may play an even more prominent role in the future.

The template‐based modeling of protein complexes represents a more complex problem than the homology modeling of individual proteins. Unlike monomeric proteins, the modeling of complexes has to deal with additional complications such as the presence of alternative intersubunit interfaces and differences in stoichiometry of homologous protein complexes.^16^, ^17^, ^18^, ^37^ In CASP14, the modeling of evolutionary nonconserved antibody‐antigen interactions was yet another example of a more complex problem. In other cases such as host‐pathogen interactions that do not always emerge from long coexistence of species, it might also be hard to apply either template‐based or coevolution‐based modeling methods.

When there are no templates and other constraints are lacking, free docking is the only feasible approach to predict the structures of protein complexes. Our CASP14 results support previous observations that docking may be successful only when subunits are sufficiently accurate.^12^, ^18^ Thus, a recent breakthrough in protein structure prediction might help not only to detect templates for multimeric structures through structure‐based searches but also to expand the applicability of protein docking. However, even if fairly accurate structures of monomers are available, the free docking is much better in predicting protein‐protein binding sites^46^, ^47^ than the exact mutual arrangement and interface contacts. This has been observed by us both in previous studies^18^ and in CASP14.

CASP14 results showed that our docking workflow still has a lot of room for improvement. With more time and more computational resources devoted for every target, some improvements could be made even while staying in the realm of rigid‐body docking and keeping our current, admittedly imperfect scoring function: (1) using a higher number of diverse input monomers, for example, generated by modeling domain motions and by remodeling flexible loops and tails; (2) ensuring that the docking software always performs a sufficiently exhaustive sampling of conformations; (3) producing structural variations of each oligomeric docking solution using molecular dynamics or other sampling techniques. These enhancements would allow to explore the conformational space more thoroughly, possibly leading to better results.^48^

Despite the limitations of our CASP14 modeling protocol, our strong performance suggests that the prediction of interchain contacts using coevolution and deep learning methods still has little impact on modeling of protein‐protein interactions. Why is that? Apparently, there are multiple reasons why interchain contact prediction is harder than intrachain. For example, contact prediction for heteromeric protein complexes requires generating joined multiple sequence alignments. The interacting proteins in the alignment are inferred by genomic distances or by phylogeny,^49^, ^50^ as well as selected using automated sequence comparison procedures.^51^ However, this significantly reduces the number of sequences in the alignment and does not guarantee the correct pairing of proteins. The alignment joining problem is not present for homo‐multimers, yet in this case the problem is to distinguish intrasubunit from intersubunit contacts.^50^ So far this problem has been solved by including the monomer structures into the prediction pipeline.^52^

In addition to the issues related to obtaining and analyzing the multiple sequence alignments, training of supervised learning‐based methods for contact prediction using the structures of protein complexes may be limited by the availability of experimental structural data. The number of possible protein complexes is believed to outnumber the number of possible protein folds,^7^, ^8^ and it is not clear whether known structures represent a significant part of all interaction types.^8^, ^53^ Moreover, there are examples of protein‐protein interactions such as antibody‐antigen or host‐pathogen protein interactions, for which principles of coevolution are hardly applicable.

Modeling of structures for individual proteins is highly automated, and multiple structure prediction servers are available in CASP^1^ and beyond.^54^ On the other hand, automated servers that predict structures of protein complexes starting from sequences are scarce. This may be not surprising bearing in mind the diversity of methods used for multimeric structure prediction. When the modeling strategy is clear, the automation becomes a software engineering task. As a result, there are servers for template‐based modeling of protein complexes, such as fully automated SWISS‐MODEL^54^ and Robetta^44^ or semiautomated PPI3D,^19^, ^55^ which we applied for homology modeling targets. Servers for protein‐protein docking are available as well.^10^ In CASP14 our free docking workflow was also automated to the point such that it could be potentially implemented as a web server. However, more complicated cases, such as modeling of large protein complexes, still require human input in merging the structures derived from different templates, combination of homology modeling with docking, and integration of diverse information from different sources.^18^

To conclude, the progress in monomeric protein structure prediction has not yet translated into similar breakthrough in structural modeling of protein complexes. A number of issues of both technical and fundamental nature have to be solved to make a leap in producing reliable structural models of protein interactions, and it will be exciting to see what developments will occur in this research area in the nearest future.

### PEER REVIEW

The peer review history for this article is available at https://publons.com/publon/10.1002/prot.26167.

## Supporting information


**Appendix** S1: Supporting information

## Data Availability

The data that support the findings of this study are openly available at the Protein Structure Prediction Center at https://predictioncenter.org/casp14.

## References

[1] Kryshtafovych A , Schwede T , Topf M , Fidelis K , Moult J . Critical assessment of methods of protein structure prediction (CASP)‐round XIII. Proteins. 2019;87(12):1011‐1020. 31589781 10.1002/prot.25823 PMC6927249

[2] Senior AW , Evans R , Jumper J , et al. Protein structure prediction using multiple deep neural networks in the 13th critical assessment of protein structure prediction (CASP13). Proteins. 2019;87(12):1141‐1148. 31602685 10.1002/prot.25834 PMC7079254

[3] Senior AW , Evans R , Jumper J , et al. Improved protein structure prediction using potentials from deep learning. Nature. 2020;577(7792):706‐710. 31942072 10.1038/s41586-019-1923-7

[4] Yang J , Anishchenko I , Park H , Peng Z , Ovchinnikov S , Baker D . Improved protein structure prediction using predicted interresidue orientations. Proc Natl Acad Sci U S A. 2020;117(3):1496‐1503. 31896580 10.1073/pnas.1914677117 PMC6983395

[5] Keskin O , Tuncbag N , Gursoy A . Predicting protein‐protein interactions from the molecular to the proteome level. Chem Rev. 2016;116(8):4884‐4909. 27074302 10.1021/acs.chemrev.5b00683

[6] Luck K , Kim D‐K , Lambourne L , et al. A reference map of the human binary protein interactome. Nature. 2020;580(7803):402‐408. 32296183 10.1038/s41586-020-2188-x PMC7169983

[7] Aloy P , Russell RB . Ten thousand interactions for the molecular biologist. Nat Biotechnol. 2004;22(10):1317‐1321. 15470473 10.1038/nbt1018

[8] Garma L , Mukherjee S , Mitra P , Zhang Y . How many protein‐protein interactions types exist in nature? PLoS One. 2012;7(6):e38913. 22719985 10.1371/journal.pone.0038913 PMC3374795

[9] Aloy P , Ceulemans H , Stark A , Russell RB . The relationship between sequence and interaction divergence in proteins. J Mol Biol. 2003;332(5):989‐998. 14499603 10.1016/j.jmb.2003.07.006

[10] Porter KA , Desta I , Kozakov D , Vajda S . What method to use for protein‐protein docking? Curr Opin Struct Biol. 2019;55:1‐7. 30711743 10.1016/j.sbi.2018.12.010 PMC6669123

[11] Lensink MF , Nadzirin N , Velankar S , Wodak SJ . Modeling protein‐protein, protein‐peptide, and protein‐oligosaccharide complexes: CAPRI 7th edition. Proteins. 2020;88(8):916‐938. 31886916 10.1002/prot.25870

[12] Lensink MF , Velankar S , Kryshtafovych A , et al. Prediction of homoprotein and heteroprotein complexes by protein docking and template‐based modeling: a CASP‐CAPRI experiment. Proteins. 2016;84(Suppl 1):323‐348. 27122118 10.1002/prot.25007 PMC5030136

[13] Lensink MF , Brysbaert G , Nadzirin N , et al. Blind prediction of homo‐ and hetero‐protein complexes: the CASP13‐CAPRI experiment. Proteins. 2019;87(12):1200‐1221. 31612567 10.1002/prot.25838 PMC7274794

[14] Lafita A , Bliven S , Kryshtafovych A , et al. Assessment of protein assembly prediction in CASP12. Proteins. 2018;86(Suppl 1):247‐256. 29071742 10.1002/prot.25408 PMC5949145

[15] Guzenko D , Lafita A , Monastyrskyy B , Kryshtafovych A , Duarte JM . Assessment of protein assembly prediction in CASP13. Proteins. 2019;87(12):1190‐1199. 31374138 10.1002/prot.25795 PMC6851419

[16] Dapkūnas J , Olechnovič K , Venclovas Č . Modeling of protein complexes in CAPRI round 37 using template‐based approach combined with model selection. Proteins. 2018;86(Suppl 1):292‐301. 28905467 10.1002/prot.25378

[17] Dapkūnas J , Kairys V , Olechnovič K , Venclovas Č . Template‐based modeling of diverse protein interactions in CAPRI rounds 38‐45. Proteins. 2020;88(8):939‐947. 31697420 10.1002/prot.25845

[18] Dapkūnas J , Olechnovič K , Venclovas Č . Structural modeling of protein complexes: current capabilities and challenges. Proteins. 2019;87(12):1222‐1232. 31294859 10.1002/prot.25774

[19] Dapkūnas J , Timinskas A , Olechnovič K , Margelevičius M , Dičiūnas R , Venclovas Č . The PPI3D web server for searching, analyzing and modeling protein‐protein interactions in the context of 3D structures. Bioinformatics. 2017;33(6):935‐937. 28011769 10.1093/bioinformatics/btw756

[20] Zimmermann L , Stephens A , Nam S‐Z , et al. A completely reimplemented MPI bioinformatics toolkit with a new HHpred server at its Core. J Mol Biol. 2018;430(15):2237‐2243. 29258817 10.1016/j.jmb.2017.12.007

[21] Söding J . Protein homology detection by HMM‐HMM comparison. Bioinformatics. 2005;21(7):951‐960. 15531603 10.1093/bioinformatics/bti125

[22] Holm L . DALI and the persistence of protein shape. Protein Sci. 2020;29(1):128‐140. 31606894 10.1002/pro.3749 PMC6933842

[23] Šali A , Blundell TL . Comparative protein modelling by satisfaction of spatial restraints. J Mol Biol. 1993;234(3):779‐815. 8254673 10.1006/jmbi.1993.1626

[24] Janson G , Grottesi A , Pietrosanto M , Ausiello G , Guarguaglini G , Paiardini A . Revisiting the “satisfaction of spatial restraints” approach of MODELLER for protein homology modeling. PLoS Comput Biol. 2019;15(12):e1007219. 31846452 10.1371/journal.pcbi.1007219 PMC6938380

[25] Zhang Y , Skolnick J . TM‐align: a protein structure alignment algorithm based on the TM‐score. Nucleic Acids Res. 2005;33(7):2302‐2309. 15849316 10.1093/nar/gki524 PMC1084323

[26] Trigg J , Gutwin K , Keating AE , Berger B . Multicoil2: predicting coiled coils and their oligomerization states from sequence in the twilight zone. PLoS One. 2011;6(8):e23519. 21901122 10.1371/journal.pone.0023519 PMC3162000

[27] Ritchie DW , Kemp GJ . Protein docking using spherical polar Fourier correlations. Proteins. 2000;39(2):178‐194. 10737939

[28] Ritchie DW , Grudinin S . Spherical polar Fourier assembly of protein complexes with arbitrary point group symmetry. J Appl Cryst. 2016;49(1):158‐167.

[29] Eastman P , Swails J , Chodera JD , et al. OpenMM 7: rapid development of high performance algorithms for molecular dynamics. PLoS Comput Biol. 2017;13(7):e1005659. 28746339 10.1371/journal.pcbi.1005659 PMC5549999

[30] Hornak V , Abel R , Okur A , Strockbine B , Roitberg A , Simmerling C . Comparison of multiple Amber force fields and development of improved protein backbone parameters. Proteins. 2006;65(3):712‐725. 16981200 10.1002/prot.21123 PMC4805110

[31] Onufriev A , Bashford D , Case DA . Exploring protein native states and large‐scale conformational changes with a modified generalized born model. Proteins. 2004;55(2):383‐394. 15048829 10.1002/prot.20033

[32] Olechnovič K , Kulberkytė E , Venclovas Č . CAD‐score: a new contact area difference‐based function for evaluation of protein structural models. Proteins. 2013;81(1):149‐162. 22933340 10.1002/prot.24172

[33] Olechnovič K , Venclovas Č . Contact area‐based structural analysis of proteins and their complexes using CAD‐score. Methods Mol Biol. 2020;2112:75‐90. 32006279 10.1007/978-1-0716-0270-6_6

[34] Olechnovič K , Venclovas Č . VoroMQA web server for assessing three‐dimensional structures of proteins and protein complexes. Nucleic Acids Res. 2019;47(W1):W437‐W442. 31073605 10.1093/nar/gkz367 PMC6602437

[35] Olechnovič K , Venclovas Č . VoroMQA: assessment of protein structure quality using interatomic contact areas. Proteins. 2017;85(6):1131‐1145. 28263393 10.1002/prot.25278

[36] Olechnovič K , Venclovas Č . Voronota: a fast and reliable tool for computing the vertices of the Voronoi diagram of atomic balls. J Comput Chem. 2014;35(8):672‐681. 24523197 10.1002/jcc.23538

[37] Bertoni M , Kiefer F , Biasini M , Bordoli L , Schwede T . Modeling protein quaternary structure of homo‐ and hetero‐oligomers beyond binary interactions by homology. Sci Rep. 2017;7(1):10480. 28874689 10.1038/s41598-017-09654-8 PMC5585393

[38] Méndez R , Leplae R , De Maria L , Wodak SJ . Assessment of blind predictions of protein‐protein interactions: current status of docking methods. Proteins. 2003;52(1):51‐67. 12784368 10.1002/prot.10393

[39] Mariani V , Biasini M , Barbato A , Schwede T . lDDT: a local superposition‐free score for comparing protein structures and models using distance difference tests. Bioinformatics. 2013;29(21):2722‐2728. 23986568 10.1093/bioinformatics/btt473 PMC3799472

[40] Zhang Y , Skolnick J . Scoring function for automated assessment of protein structure template quality. Proteins. 2004;57(4):702‐710. 15476259 10.1002/prot.20264

[41] Mukherjee S , Zhang Y . MM‐align: a quick algorithm for aligning multiple‐chain protein complex structures using iterative dynamic programming. Nucleic Acids Res. 2009;37(11):e83. 19443443 10.1093/nar/gkp318 PMC2699532

[42] Sela‐Culang I , Kunik V , Ofran Y . The structural basis of antibody‐antigen recognition. Front Immunol. 2013;4:302. 24115948 10.3389/fimmu.2013.00302 PMC3792396

[43] Grinter R , Morris FC , Dunstan RA , et al. BonA from Acinetobacter baumannii forms a divisome‐localized decamer that supports outer envelope function. bioRxiv. 2020. 10.1101/2020.09.01.278697. PMC8406262 34311571

[44] Song Y , DiMaio F , Wang RY‐R , et al. High‐resolution comparative modeling with RosettaCM. Structure. 2013;21(10):1735‐1742. 24035711 10.1016/j.str.2013.08.005 PMC3811137

[45] Altschul SF , Madden TL , Schäffer AA , et al. Gapped BLAST and PSI‐BLAST: a new generation of protein database search programs. Nucleic Acids Res. 1997;25(17):3389‐3402. 9254694 10.1093/nar/25.17.3389 PMC146917

[46] Fernández‐Recio J , Totrov M , Abagyan R . Identification of protein‐protein interaction sites from docking energy landscapes. J Mol Biol. 2004;335(3):843‐865. 14687579 10.1016/j.jmb.2003.10.069

[47] Viswanathan R , Fajardo E , Steinberg G , Haller M , Fiser A . Protein‐protein binding supersites. PLoS Comput Biol. 2019;15(1):e1006704. 30615604 10.1371/journal.pcbi.1006704 PMC6336348

[48] Kurkcuoglu Z , Bonvin AMJJ . Pre‐ and post‐docking sampling of conformational changes using ClustENM and HADDOCK for protein‐protein and protein‐DNA systems. Proteins. 2020;88(2):292‐306. 31441121 10.1002/prot.25802 PMC6973081

[49] Zeng H , Wang S , Zhou T , et al. ComplexContact: a web server for inter‐protein contact prediction using deep learning. Nucleic Acids Res. 2018;46(W1):W432‐W437. 29790960 10.1093/nar/gky420 PMC6030867

[50] Szurmant H , Weigt M . Inter‐residue, inter‐protein and inter‐family coevolution: bridging the scales. Curr Opin Struct Biol. 2018;50:26‐32. 29101847 10.1016/j.sbi.2017.10.014 PMC5940578

[51] Faure G , Andreani J , Guerois R . InterEvol database: exploring the structure and evolution of protein complex interfaces. Nucleic Acids Res. 2012;40:D847‐D856. 22053089 10.1093/nar/gkr845 PMC3245184

[52] Uguzzoni G , John Lovis S , Oteri F , Schug A , Szurmant H , Weigt M . Large‐scale identification of coevolution signals across homo‐oligomeric protein interfaces by direct coupling analysis. Proc Natl Acad Sci U S A. 2017;114(13):E2662‐E2671. 28289198 10.1073/pnas.1615068114 PMC5380090

[53] Ahnert SE , Marsh JA , Hernández H , Robinson CV , Teichmann SA . Principles of assembly reveal a periodic table of protein complexes. Science. 2015;350(6266):aaa2245. 26659058 10.1126/science.aaa2245

[54] Waterhouse A , Bertoni M , Bienert S , et al. SWISS‐MODEL: homology modelling of protein structures and complexes. Nucleic Acids Res. 2018;46(W1):W296‐W303. 29788355 10.1093/nar/gky427 PMC6030848

[55] Dapkūnas J , Venclovas Č . Template‐based modeling of protein complexes using the PPI3D web server. Methods Mol Biol. 2020;2165:139‐155. 32621223 10.1007/978-1-0716-0708-4_8

