# Supplementary material for: Modeling of protein complexes in CASP14 with emphasis on the interaction interface prediction
Source: Proteins. 2021 Jul 5;89(12):1834–43. doi: 10.1002/prot.26167 (PMC9292421; doi:10.1002/prot.26167)
Supplement: Supplementary file 1 — Appendix S1: Supporting information [file PROT-89-1834-s001.pdf]

Modeling of protein complexes in CASP14 with emphasis on the interaction interface prediction

Justas Dapkūnas, Kliment Olechnovič and Česlovas Venclovas

Institute of Biotechnology, Life Sciences Center, Vilnius University, Saulėtekio 7, LT-10257 Vilnius, Lithuania

Supplementary information

Corresponding author:

Česlovas Venclovas
Institute of Biotechnology, Life Sciences Center
Vilnius University
Saulėtekio 7,
LT-10257 Vilnius, Lithuania

Phone: +370-5-223-4368
E-mail: ceslovas.venclovas@bti.vu.lt

Supplementary Methods

VoroMQA-dark method for structure quality assessment

VoroMQA-dark method for model accuracy estimation uses a feed-forward neural network (NN) trained to predict local (per-residue) CAD-score[1] values. The training and cross-validation was done using 28377 models of 199 targets from CASP 8-13.

The targeted NN output for each residue is a vector of three local and semi-local CAD-score values:

- CAD-score(depth=0), based on all the inter-residue contacts involving the central residue;
- CAD-score(depth=1), based on all the inter-residue contacts involving at least one residue from the first layer of neighbors (the direct neighbors) of the central residue;
- CAD-score(depth=2), based on all the inter-residue contacts involving at least one residue from the first two layers of neighbors (the direct neighbors and the neighbors of the direct neighbors) of the central residue.

The NN input vector consists of 299 values describing a protein residue and its environment. The first 20 values are used to describe the residue type using one-hot encoding. The remaining 279 input vector values describe the residue environment and are derived from the Voronoi tessellation-based inter-atom contact areas and the corresponding contact potential values - the same pseudo-energy potential values as in the original VoroMQA method (VoroMQA-light)[2]. The environment descriptors are summed contact areas, summed atomic volumes, and pseudo-energy values (both raw and normalized by the corresponding summed contact area or summed atomic volume). The descriptors are computed for three layers of residue neighborhoods using tessellation breadth-first search and accumulating convolution operations.

As the input vector is "pre-convoluted", convolutional layers were not used in the NN architecture. The final architecture was determined through an iterative five-fold cross-validation process, monitoring the Pearson correlation of predicted and actual local scores. Data splitting for cross validation was performed on the level of CASP targets. The final NN architecture has only one fully-connected hidden layer of 24 neurons.

The cross-validation results showed that the CAD-score(depth=2) local scores were the most predictable, the average Pearson correlation coefficients between the predicted and the actual local scores were

- 0.81 for depth=2,
- 0.77 for depth=1,
- 0.65 for depth=0.

However, surprisingly, the best estimate for the global CAD-score was the average predicted CAD-score (depth=0). The average Spearman correlation coefficients between the estimated and the actual global scores were

- 0.79 for depth 0,
- 0.77 for depth 1,
- 0.77 for depth 2.

Therefore, the VoroMQA-dark global structure score is computed by averaging the predicted CAD-score (depth=0) values.

Using VoroMQA-dark for scoring and ranking complexes

For a multi-chain structure, VoroMQA-dark can produce two scores:

1. VoroMQA-dark global score, which is an average of the predicted local (per-residue) scores;
2. Inter-chain interface energy score, the same as in the VoroMQA-light method.

The global score and the interface energy score are used to perform a tournament-based ranking of structural models[3, 4]. For every model A in a given set of models we counted "wins" and "draws". A win for model A is when another model B in the set is inferior to A according to both scores. A draw is when B is neither inferior nor superior to A, which happens when the two scores disagree. The models are ranked according to their numbers of wins and draws.

An important aspect of the tournament ranking scheme is the usage of non-strict comparisons. A tolerance value t is used when checking if the global VoroMQA-dark score s(A) of model A is greater than the score s(B) of model B. When checking for a win of model A against model B, s(A) is considered to be superior to s(B) if (s(A)+t)>s(B). Thus, model A can win against model B even when s(A)<s(B), if the interface energy score of A is better. Therefore, increasing a tolerance value for comparing global scores is a way to put more emphasis on the interface energy score. Based on the testing with the CASP13 data, different tolerance values were used depending on the structure type:

- t=0.01 for homo-oligomers;
- t=0.02 for hetero-oligomers.

VoroMQA interface energy values were compared without any tolerance.

The new VoroMQA-dark-based model selection protocol, called "VoroMQA-select-new", did not perform better than the top 2 human groups overall. However, our new model selection protocol performed consistently better than the VoroMQA-light-based method we used in CASP13[4] ("VoroMQA-select-old"), as shown in Supplementary Figure S10.

Supplementary Tables

Supplementary Table S1. The quality evaluation of our best models for CASP14 targets. Best model is the model that has the largest sum of z-scores for ICS, IPS, lDDT and TM-score.

Target	Oligomeric state	Difficulty[1]	Modeling strategy[2]	QS-score category	QS-score	ICS	IPS	lDDT	TM-score
T1032	A2	Easy	CM	Medium	0.56	0.45	0.53	0.55	0.64
T1034	A4	Medium	CM	Incorrect	0.01	0.01	0.08	0.22	0.24
H1036	A3B3C3	Medium	CM	Medium	0.61	0.53	0.61	0.61	0.65
H1036v0	ABC	Medium	CM	Medium	0.31	0.31	0.46	0.63	0.64
T1038	A2	Hard	FD	Incorrect	0.08	0.07	0.11	0.23	0.22
H1045	A1B1	Medium	CM	High	0.84	0.78	0.80	0.74	0.87
H1047	(A1B1)x	Hard	FD	Incorrect	0.00	0.00	0.09	0.61	0.37
T1048	A4	Medium	CC	Incorrect	0.05	0.05	0.32	0.35	0.73
T1052	A3	Easy	CM	Medium	0.67	0.64	0.64	0.60	0.74
T1054	A2	Hard	FD	Low	0.12	0.02	0.50	0.62	0.55
H1060	*A6B3C12D6:*								
H1060v1	A2B1C1D1	Medium	H	Low	0.14	0.08	0.25	0.63	0.32
H1060v2	A3	Medium	CM	Medium	0.38	0.22	0.55	0.70	0.91
H1060v3	A3	Medium	CM	Medium	0.44	0.28	0.56	0.53	0.84
H1060v4	A12	Medium	TBD	Medium	0.38	0.26	0.39	0.54	0.83
H1060v5	A6	Medium	TBD	Medium	0.48	0.41	0.48	0.66	0.80
T1061	A3	Hard	H	Low	0.20	0.19	0.20	0.36	0.45
H1065	A1B1	Hard	FD	Low	0.10	0.04	0.57	0.62	0.61
T1070	A3	Hard	H	Low	0.17	0.11	0.19	0.41	0.36
H1072	A2B2	Medium	CC	Low	0.29	0.21	0.31	0.49	0.44
T1078	A2	Medium	H	Low	0.15	0.12	0.33	0.54	0.63
T1080	A3	Hard	H	Low	0.24	0.20	0.42	0.23	0.46
H1081	*A20:*								
H1081v0	A0	Medium	FD	Medium	0.62	0.24	0.30	0.81	0.97
T1083	A2	Medium	H	Medium	0.64	0.57	0.60	0.55	0.70
T1084	A2	Medium	H	High	0.86	0.63	0.63	0.76	0.89
T1087	A2	Medium	H	Medium	0.44	0.26	0.59	0.48	0.59
H1097	A1B1C1D1E1	Medium	H	Medium	0.38	0.30	0.42	0.42	0.68
T1099	*A240:*								
T1099v0	A4	Medium	CM	Incorrect	0.09	0.06	0.31	0.39	0.59
T1099v1	A2	Medium	CM	Incorrect	0.03	0.01	0.33	0.40	0.59
T1099v2	A2	Medium	CM	Medium	0.59	0.39	0.45	0.45	0.60

[1] Target difficulty categories were assigned by CASP assessors.
[2] Abbreviations of modeling strategies: CM – comparative modeling, FD – free docking, CC – coiled-coil modeling, H – hybrid approach, TBD – template-based docking.

Supplementary Table S2. Evaluation of interaction interfaces in our H1097 models, showing that interactions with chain E are predicted worse than other interactions. Interface CAD-score is an analog of ICS, site CAD-score is analog of IPS.

H1097 interface		Area, Å^2	Model 1 (template: 5zc3)		Model 2 (template: 6pst)	
			Interface CAD-score	Site CAD-score	Interface CAD-score	Site CAD-score
Inter-chain		19240	0.22	0.47	0.10	0.42
ABCD inter-chain		14940	0.27	0.53	0.12	0.47
A	B	3197	0.21	0.63	0.24	0.61
A	D	4726	0.42	0.61	0.12	0.60
A	E	2453	0.05	0.13	0.07	0.27
B	C	675	0.52	0.62	0.11	0.63
B	D	3044	0.20	0.37	0.09	0.36
C	D	3288	0.12	0.33	0.02	0.25
C	E	335	0.00	0.40	0.00	0.07
D	E	1512	0.00	0.09	0.00	0.15
ABCD	E	4299	0.03	0.14	0.04	0.21

Supplementary Table S3. The accuracy of our docking models, having the largest ICS or largest IPS scores.

Target	Model[1]	QS-score	ICS	IPS	s1_lDDT	s1_TM-score	s2_lDDT	s2_TM-score
T1038o	1	0.01	0.01	0.12	0.38	0.31	0.38	0.31
T1038o	3	0.07	0.06	0.10	0.25	0.29	0.25	0.29
H1047	2	0.00	0.00	0.09	0.55	0.38	0.68	0.56
T1054o	4	0.12	0.02	0.50	0.74	0.84	0.74	0.84
H1065	1	0.10	0.04	0.57	0.81	0.93	0.68	0.82
H1065	2	0.06	0.01	0.60	0.76	0.87	0.72	0.85
H1081v0	3	0.42	0.30	0.37	0.84	0.98	0.84	0.98
T1083o	4	0.64	0.57	0.60	0.61	0.70	0.61	0.70
T1084o	1	0.86	0.63	0.63	0.80	0.87	0.80	0.87
T1087o	2	0.32	0.30	0.45	0.61	0.74	0.61	0.74
T1087o	4	0.30	0.27	0.49	0.47	0.56	0.47	0.56

[1] Models with the largest differences between IPS and ICS are shaded

Supplementary Figures

Supplementary Figure S1. Comparative modeling workflow in CASP14. Methods developed in our laboratory are colored blue.

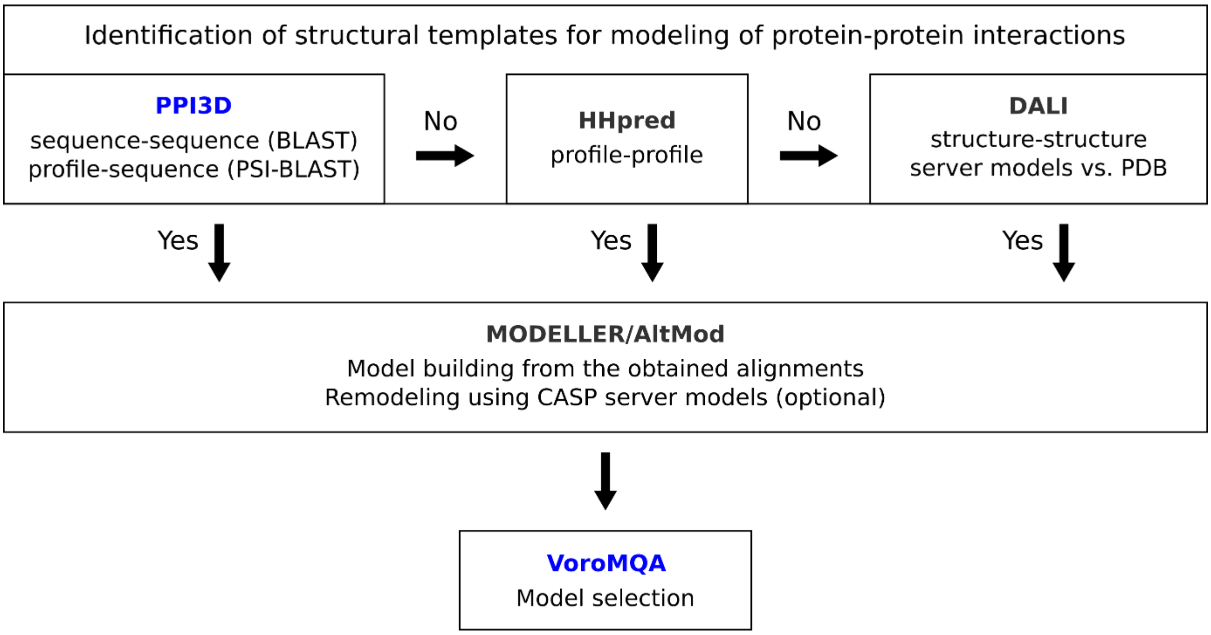

Supplementary Figure S2. Free docking workflow in CASP14. Methods developed in our laboratory are colored blue.

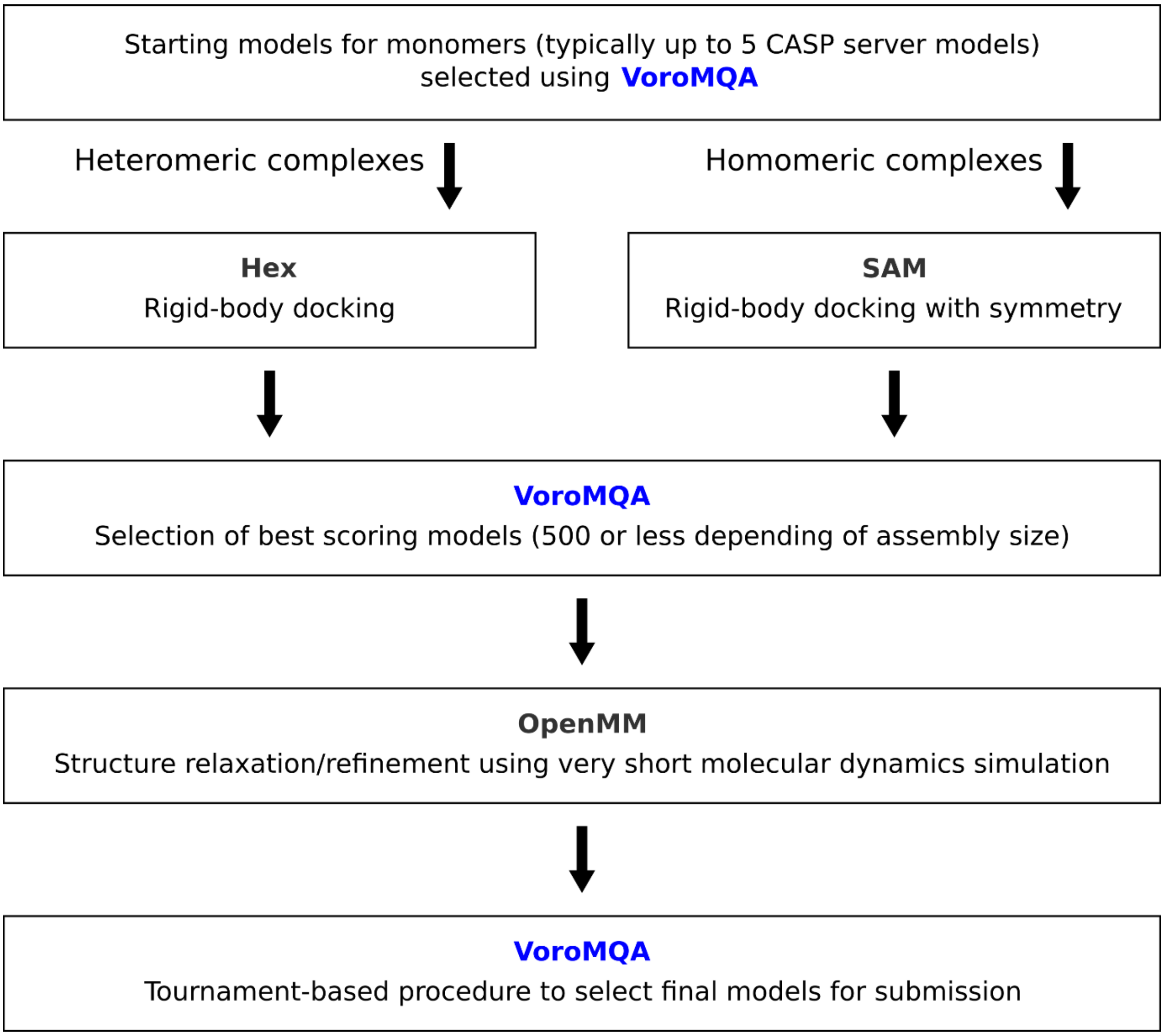

Supplementary Figure S3. Cumulative z-score (left side) and raw score (right side) values of the models designated as first. Targets were ordered by the maximum achieved ICS score.

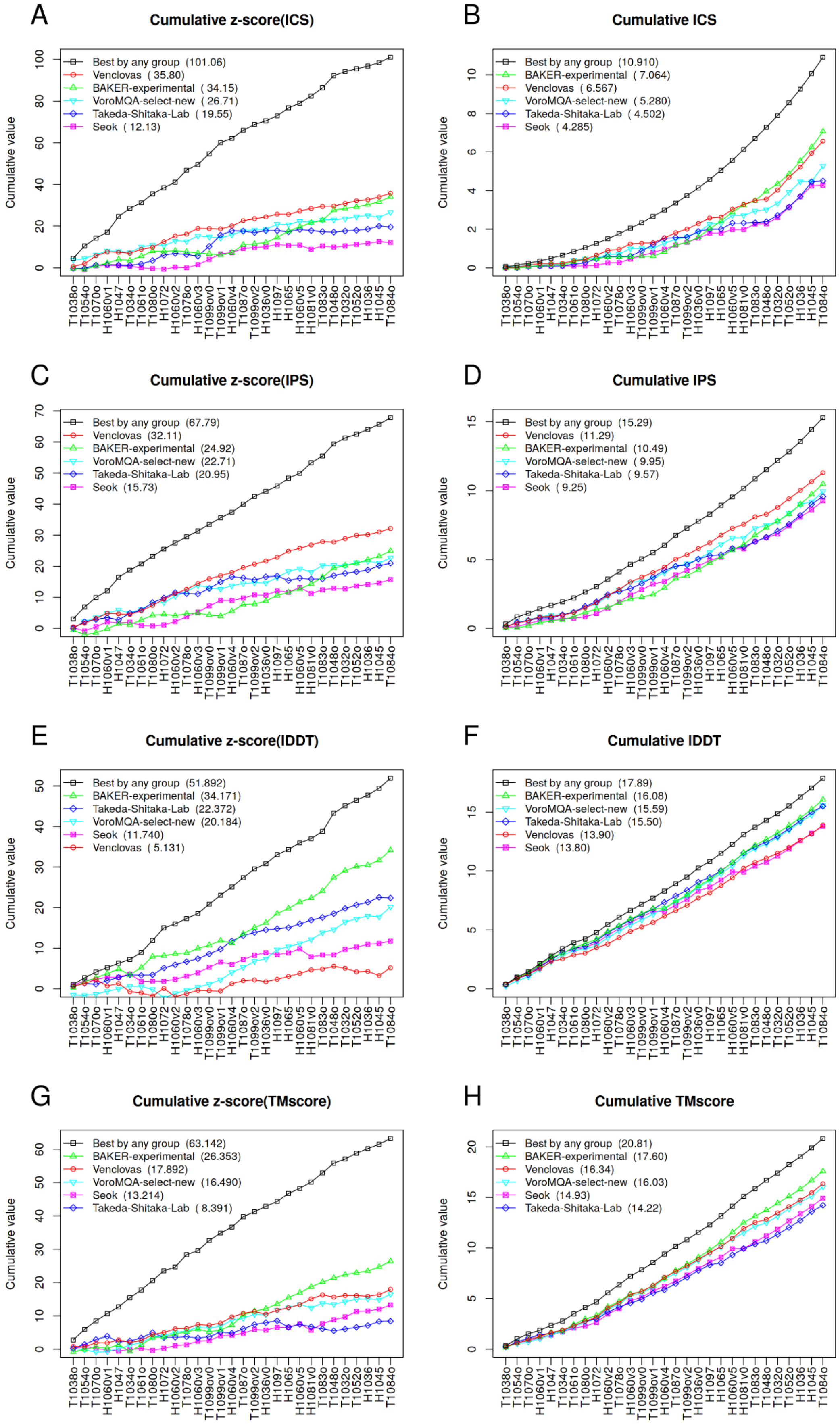

Supplementary Figure S4. Examples of model pairs with large differences of lDDT-oligo z-scores, but not of absolute lDDT-oligo values. The models on the left (produced by the "Venclovas" group), have lower lDDT-oligo z-score and absolute values than the models on the right (produced by other groups). Despite that, the models on the left feature highly accurate intersubunit interfaces as indicated by high ICS and TM-score values, whereas the models on the right have largely incorrect interfaces (ICS values are close to zero).

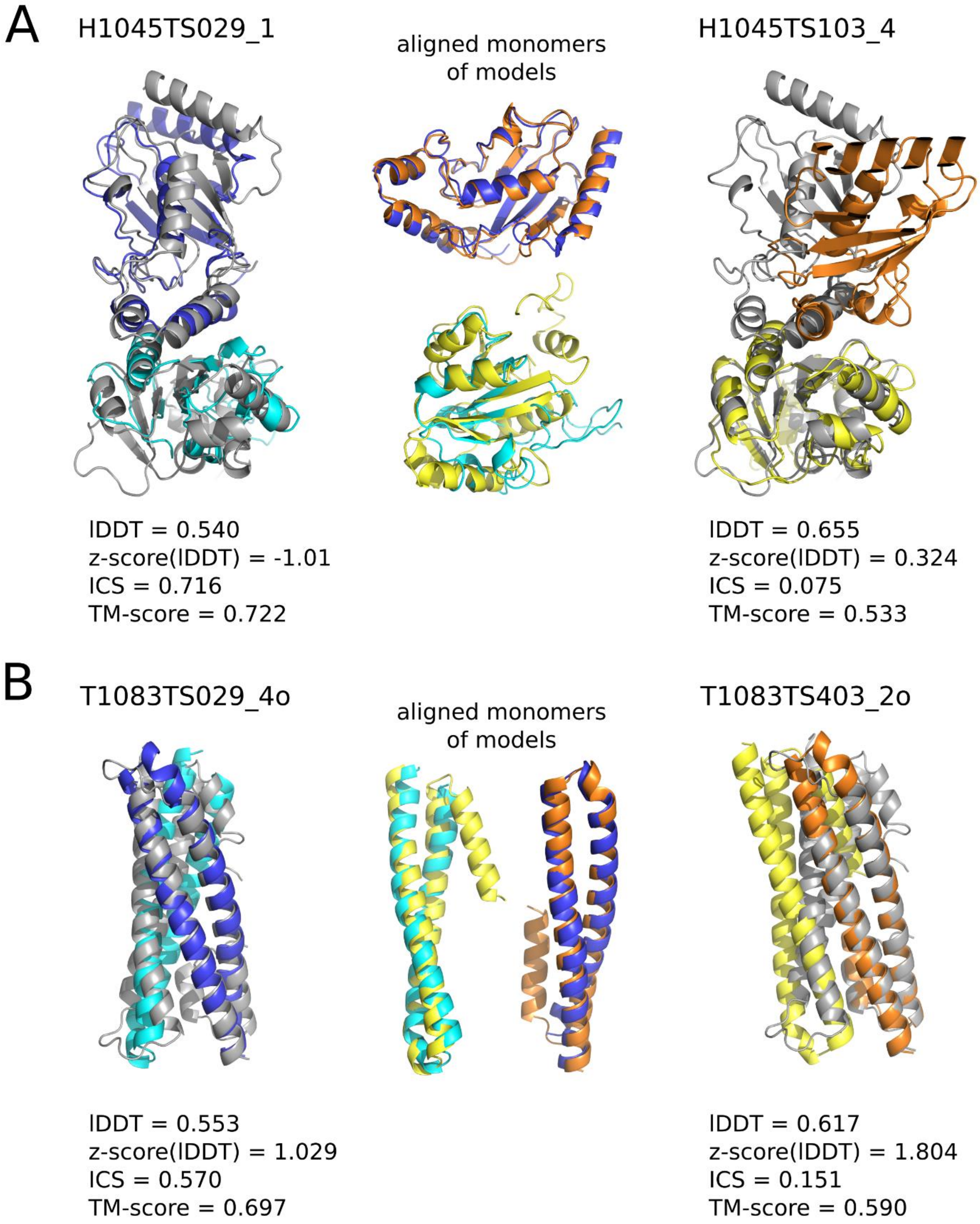

Supplementary Figure S5. Cumulative CAD-score-based z-score (left side) and raw score (right side) values of the models designated as first. Targets were ordered by the maximum achieved ICS score.

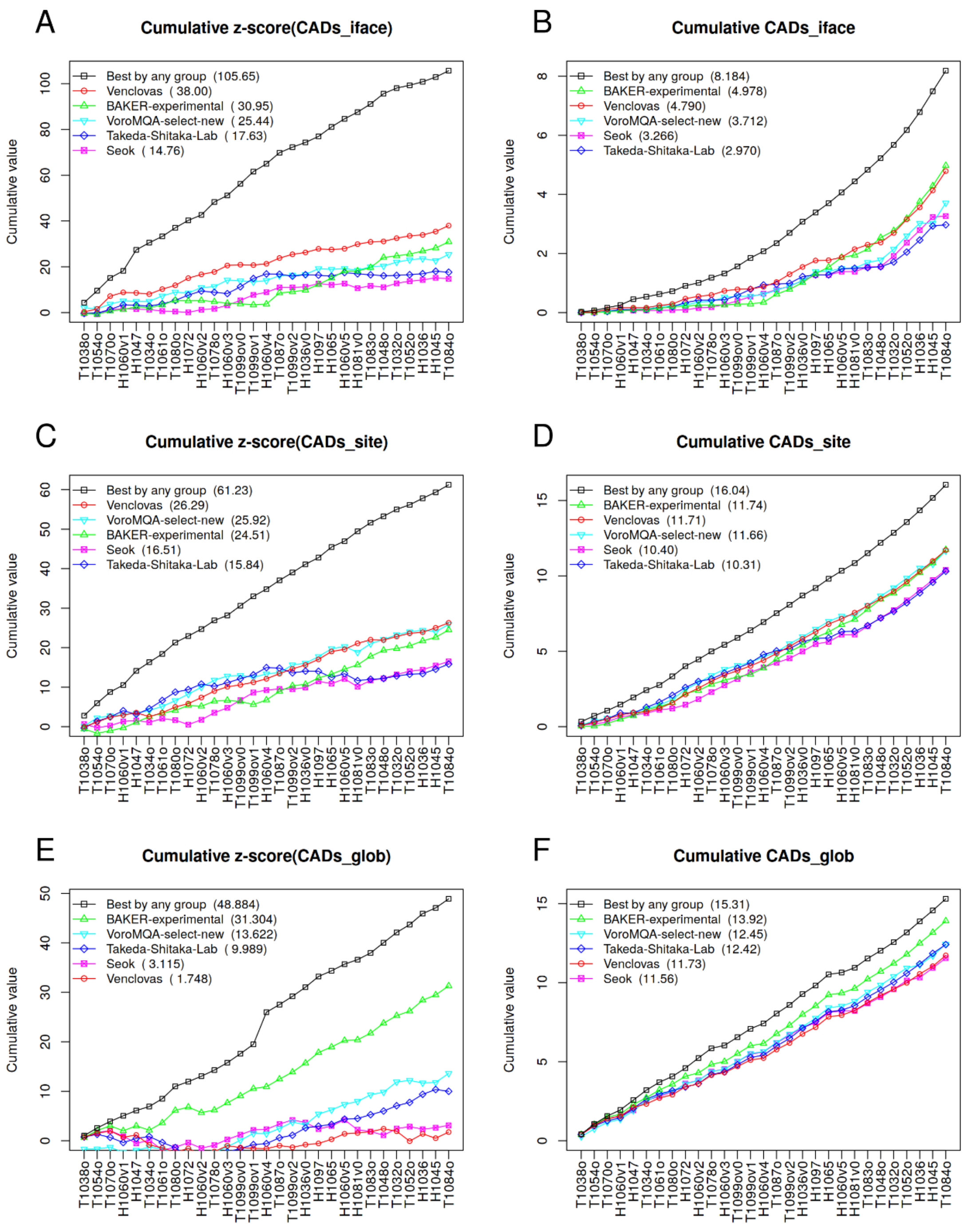

Supplementary Figure S6. Cumulative combined z-score values of the models designated as first. Targets were ordered by the maximum achieved ICS score.

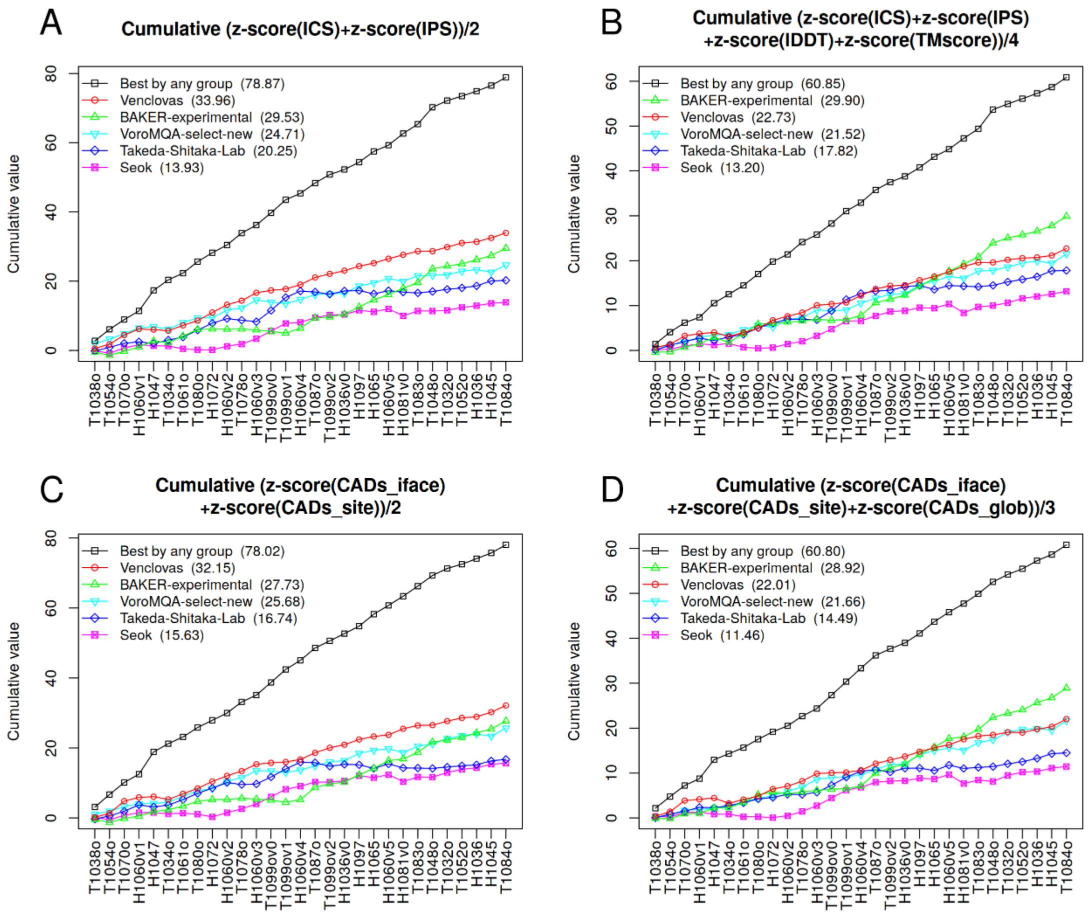

Supplementary Figure S7. Cumulative combined z-score values of the models with the best scores. Targets were ordered by the maximum achieved ICS score.

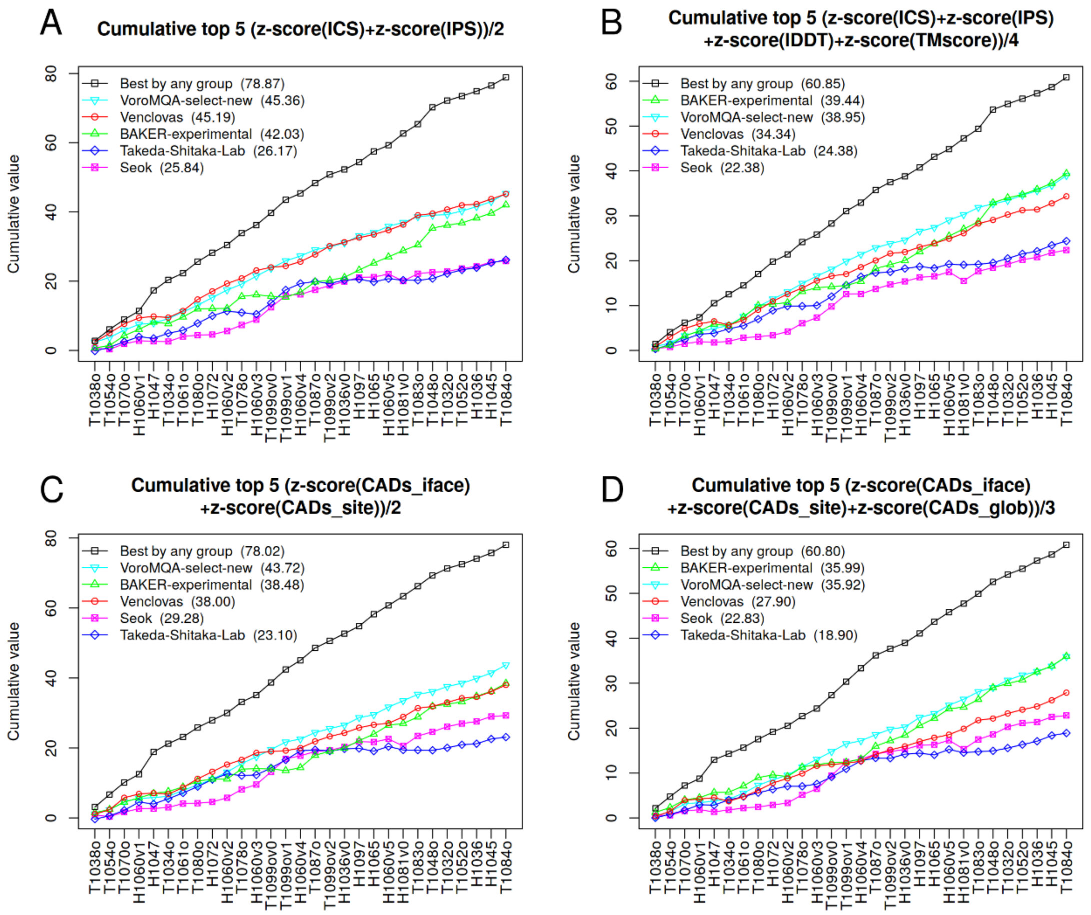

Supplementary Figure S8. In our model (colored) of H1036 (grey, PDB: 6VN1), antibody (red) is bound to a different epitope of viral trimer (green). Structure superposition is done by aligning the first chain (green) using TM-align[5].

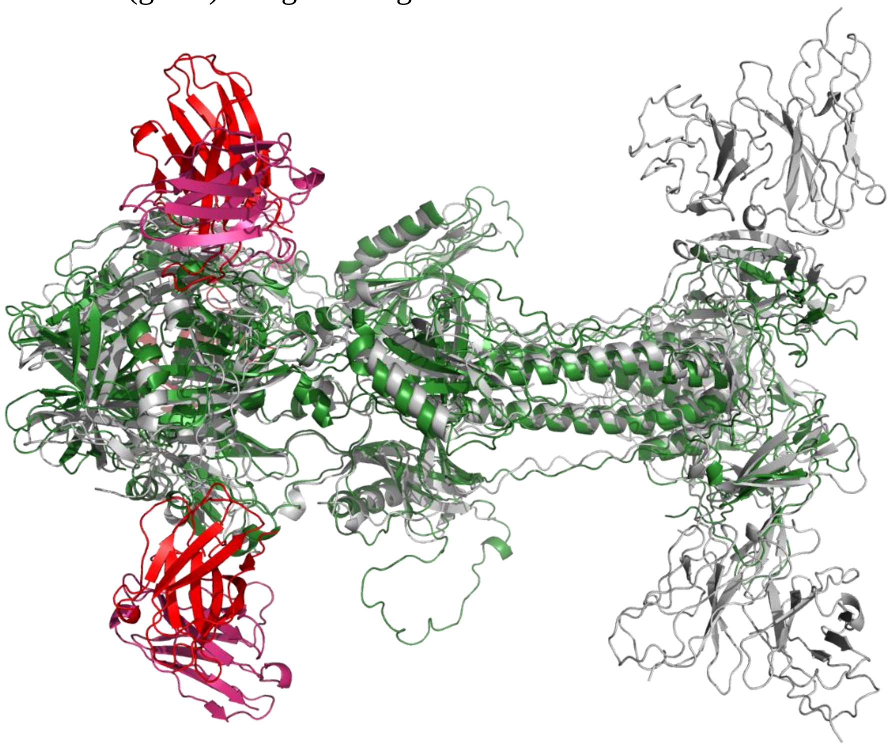

Supplementary Figure S9. Models of different quality for two interfaces of T1099 (PDB: 6YGH), based on a target-template HHpred alignment with large gaps in the interface region. Target structures are grey, model structures are in different colors. Structure superposition was done by aligning the first chain (green) using TM-align[5], sequence alignment was visualised using Jalview[6].

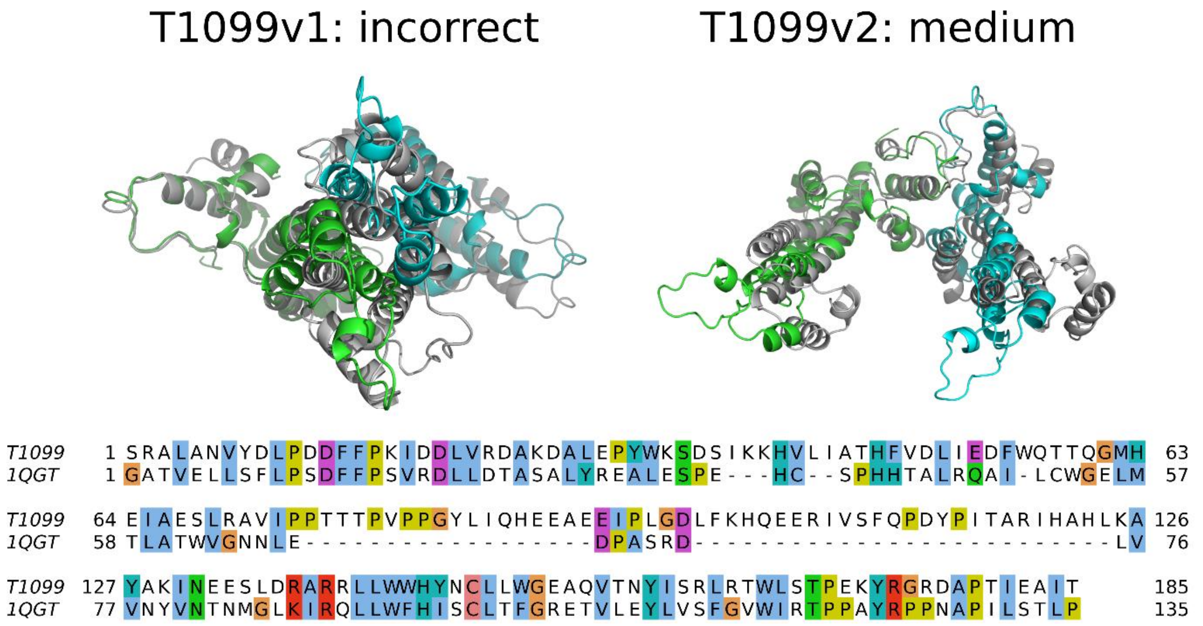

Supplementary Figure S10. Cumulative z-score values achieved by several VoroMQA-based ranking methods applied to the full set of models from all the groups. Targets were ordered by the maximum achieved ICS score. "VoroMQA-select-new-tol0" is a variation of "VoroMQA-select-new" that does not use tolerances for full-structure scores.

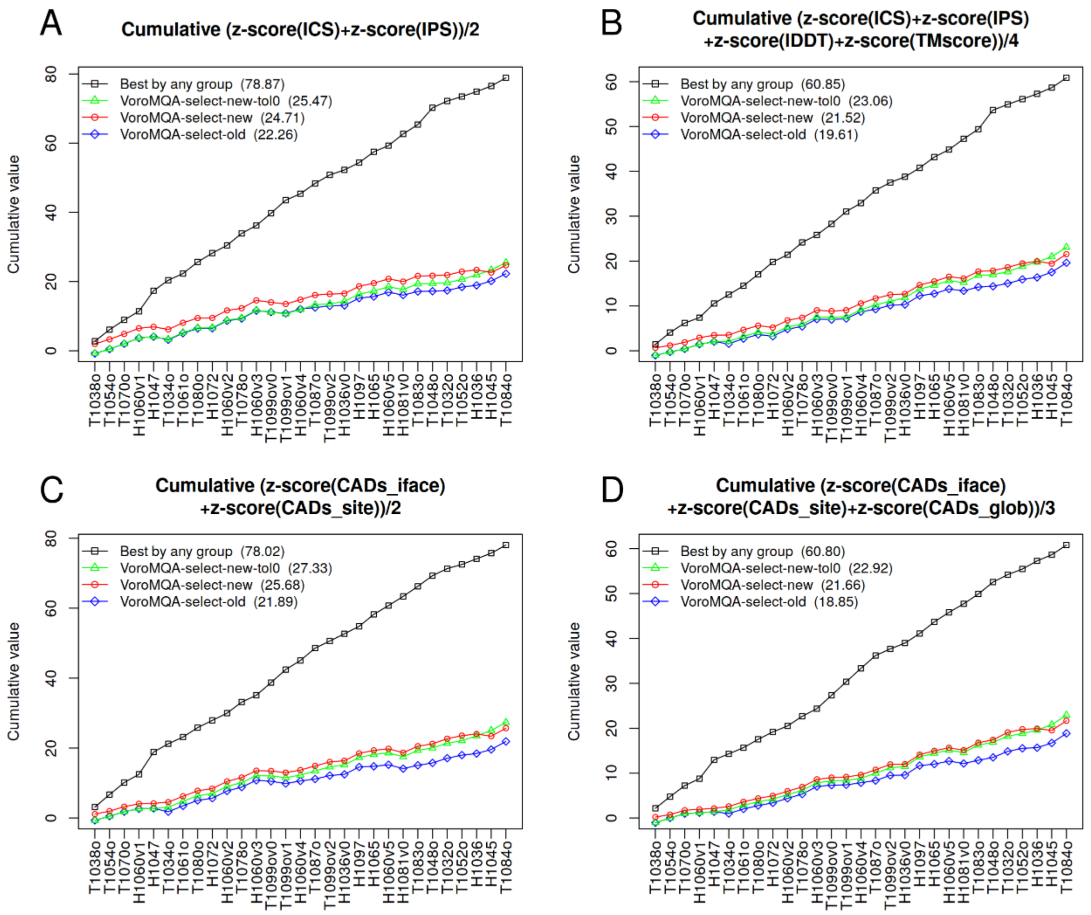

Supplementary References

1. Olechnovič K, Kulberkytė E, Venclovas Č. CAD-score: a new contact area difference-based function for evaluation of protein structural models. Proteins 2013;81(1):149–162.
2. Olechnovič K, Venclovas Č. VoroMQA: Assessment of protein structure quality using interatomic contact areas. Proteins 2017;85(6):1131–1145.
3. Dapkūnas J, Olechnovič K, Venclovas Č. Modeling of protein complexes in CAPRI Round 37 using template-based approach combined with model selection. Proteins 2018;86 Suppl 1:292–301.
4. Dapkūnas J, Olechnovič K, Venclovas Č. Structural modeling of protein complexes: Current capabilities and challenges. Proteins 2019;87(12):1222–1232.
5. Zhang Y, Skolnick J. TM-align: a protein structure alignment algorithm based on the TM-score. Nucleic Acids Res 2005;33(7):2302–2309.
6. Waterhouse AM, Procter JB, Martin DMA, Clamp M, Barton GJ. Jalview Version 2--a multiple sequence alignment editor and analysis workbench. Bioinformatics 2009;25(9):1189–1191.
